# Derivation and validation of clinical phenotypes for COPD: a systematic review

**DOI:** 10.1186/s12931-015-0208-4

**Published:** 2015-04-18

**Authors:** Lancelot M Pinto, Majed Alghamdi, Andrea Benedetti, Tasneem Zaihra, Tara Landry, Jean Bourbeau

**Affiliations:** Respiratory Division, McGill University Health Centre, Montreal, Quebec Canada; Respiratory Epidemiology and Clinical Research Unit, Montreal Chest Institute, McGill University Health Centre, Montreal, Quebec Canada; Department of Epidemiology, Biostatistics & Occupational Health, Montreal, Quebec Canada; School of PH & OT, Faculty of Medicine, McGill University, Quebec, Canada; Division of Clinical Epidemiology, McGill University Health Centre, Montreal, Quebec Canada; Medical Library, Montreal General Hospital, McGill University Health Centre, Montreal, Quebec Canada; Montreal Chest Institute, McGill University Health Centre, 3650 St.Urbain, Room K1.32, H2X 2P4 Montréal (Québec), Canada

**Keywords:** COPD phenotypes, Phenotypes, Cluster analysis, Systematic review

## Abstract

**Background:**

The traditional classification of COPD, which relies solely on spirometry, fails to account for the complexity and heterogeneity of the disease. Phenotyping is a method that attempts to derive a single or combination of disease attributes that are associated with clinically meaningful outcomes. Deriving phenotypes entails the use of cluster analyses, and helps individualize patient management by identifying groups of individuals with similar characteristics. We aimed to systematically review the literature for studies that had derived such phenotypes using unsupervised methods.

**Methods:**

Two independent reviewers systematically searched multiple databases for studies that performed validated statistical analyses, free of definitive pre-determined hypotheses, to derive phenotypes among patients with COPD. Data were extracted independently.

**Results:**

9156 citations were retrieved, of which, 8 studies were included. The number of subjects ranged from 213 to 1543. Most studies appeared to be biased: patients were more likely males, with severe disease, and recruited in tertiary care settings. Statistical methods used to derive phenotypes varied by study. The number of phenotypes identified ranged from 2 to 5. Two phenotypes, with poor longitudinal health outcomes, were common across multiple studies: young patients with severe respiratory disease, few cardiovascular co-morbidities, poor nutritional status and poor health status, and a phenotype of older patients with moderate respiratory disease, obesity, cardiovascular and metabolic co-morbidities.

**Conclusions:**

The recognition that two phenotypes of COPD were often reported may have clinical implications for altering the course of the disease. This review also provided important information on limitations of phenotype studies in COPD and the need for improvement in future studies.

**Electronic supplementary material:**

The online version of this article (doi:10.1186/s12931-015-0208-4) contains supplementary material, which is available to authorized users.

## Background

Chronic Obstructive Pulmonary Disease (COPD) is the 4^th^ leading cause of mortality worldwide, and causes significant morbidity [1,2]. Criteria have been developed to diagnose and grade the severity of COPD based on the post-bronchodilator FEV_1_ [3]. However, these criteria fail to account for the complexity and heterogeneity of the disease, variable symptomatic manifestations, progression and prognosis of the disease. With progress in the understanding of the disease, and developments in the fields of radiology, genetics, and statistical analyses, identifying phenotypes of the disease with the aim of individualized treatment has now gained significance.

A COPD phenotype has been defined as “A single or combination of disease attributes that describe differences between individuals with COPD as they relate to clinically meaningful outcomes (symptoms, exacerbations, response to treatment, speed of progression of the disease or death)” [4]. Identifying such phenotypes entails use of statistical techniques such as cluster analysis [5]. The goal of cluster analysis is to assign subjects to groups, where subjects in the same cluster are more similar to each other than they are to subjects in other groups.

This review systematically searches the available literature to identify studies that have derived clinical phenotypes in COPD using validated statistical methods, free of definitive pre-determined hypotheses. We aimed to summarize such studies, and the robustness of findings, by identifying the combination of disease attributes in patients with COPD, over and above the traditional spirometric classification of disease, that describe differences between individuals as they relate to clinically meaningful outcomes. We also aimed to identify the limitations of the studies and what needed to be done to improve this field of research.

## Methods

A detailed protocol was written prior to starting the review, which identified the key question, and the criteria for the systematic review. We used the Preferred Reporting Items for Systematic Reviews and Meta-Analysis statement [6] as the template for reporting the review.

We searched the literature for studies that enrolled patients with COPD, as defined by GOLD criteria [3], in which unsupervised (hypothesis-free) cluster analyses were used to derive clinical phenotypes as they related to clinically meaningful outcomes.

### Search strategy

The following databases were searched for relevant studies: MEDLINE (via OvidSP 1946 to 22/Apr/2013; via PubMed 1946 to 22/Apr/2013); Embase Classic + Embase (via OvidSP 1947 to 19/Apr/2013); BIOSIS Previews (via OvidSP 1969 to 2013 Week 20); Web of Science (via ThomsonReuters 1996 to 22/Apr/2013); Scopus (via Elsevier 1996 to 22/Apr/2013); CENTRAL (via Cochrane Library). The search strategy used text words and relevant indexing to answer the following question: What are the different phenotypes of COPD that have been derived, based on subject characteristics? The full MEDLINE strategy (Appendix 1) was applied to all databases, with modifications to search terms as necessary. Further studies were identified in Web of Science and Scopus (02/Oct/2013) by carrying out by citations searches for studies citing included studies, as well as by examining their reference lists. The Medline strategy was rerun prior to submission (two relevant studies were found, one of which was included).

### Selection of studies

We selected studies that included at least 50 patients who were 18 years and above, and in which a statistical method was used to identify clusters of subjects. We excluded abstracts, reviews and commentaries. Studies that exclusively enrolled subjects with alpha-1 antitrypsin deficiency or those that tested single risk factors, including genetic polymorphisms, for association with outcomes in COPD were excluded. Studies that tested empirically defined phenotypes without an analytical justification of these phenotypes, and those that did not analyze the association of the derived clusters with clinically meaningful outcomes were also excluded.

### Data extraction and consensus

Two reviewer authors (LMP and MA) independently scrutinized titles and abstracts for eligibility. Citations deemed relevant by either reviewer were selected and papers retrieved for full-text review. Each eligible article was independently assessed by two reviewers (LMP and MA). Disagreements were resolved by discussion between the reviewers. Study quality was assessed using the Strengthening the Reporting of Observational studies in Epidemiology (STROBE) checklist [7].

The two reviewer authors (LMP and MA) independently extracted data from each study using a data extraction form. Disagreements were resolved by discussion. Data were extracted for various characteristics, including the following: author, publication year, study design, number of participants, criteria for inclusion and exclusion, statistical methods for cluster analysis and outcomes assessed.

### Statistical analysis

Descriptive statistics for the clinical features most commonly used in the routine care of COPD patients were collected and tabulated from the included studies. Due to significant heterogeneity in selection of patients, cluster analyses and derived phenotypes, pooling of results was not deemed appropriate.

## Results

### Studies selected for the systematic review

**Figure 1:**
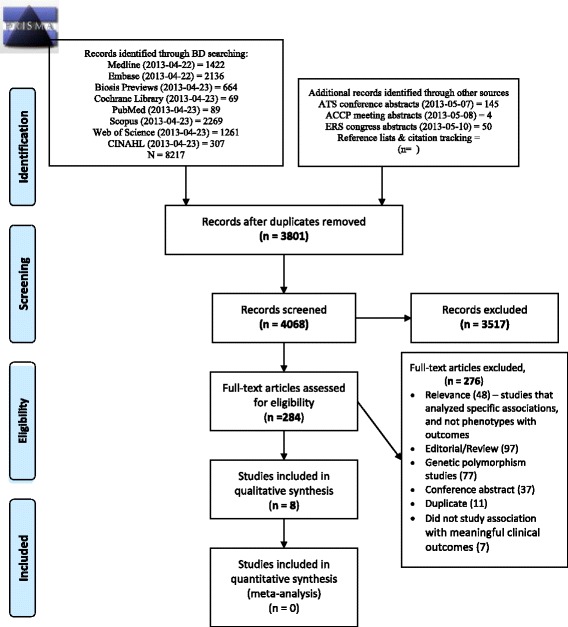
PRISMA flow diagram. From: Moher D, Liberati A, Tetzlaff J, Altman DG, The PRISMA Group (2009). Preferred Reporting Items for Systematic Reviews and Meta-Analyses: The PRISMA Statement. PLoS Med 6(6): e1000097. doi:10.1371/journal.pmed1000097. For more information, visit www.prisma-statement.org.

We identified 9156 citations, of which 4068 unique published articles were identified after exclusion of duplicate articles. After screening titles and abstracts, 284 studies were found to satisfy the criteria for further review and their full-texts were retrieved. After full-text review, 276 studies were excluded for various reasons, and 8 studies (7 observational studies, one study with data pooled from two randomized controlled trials) were included in the systematic review. Figure 1 summarizes the selection criteria for the included studies.

In the 8 studies identified [8-15], all conducted in Europe and USA, the median sample size included in the analyses was 332 subjects (range 213-1543). Of the 7 studies that described the setting, only one study had a subset of its enrolled subjects recruited from a community-based lung cancer screening study [10]; all the other studies were conducted in a university-based tertiary care setting. A summary of the study characteristics of the included studies can be found in Table 1; a more detailed description can be found in the online supplement, Additional file 1: Table S1.

**Table 1:** **Characteristics of the included studies of clinical phenotypes for COPD**

**Study, country, period**	**Setting, design**	**Inclusion criteria**	**Number included in the analysis/ number eligible**	**Reason for exclusion**	**Difference between included and excluded patients**
Burgel et al. (2010), France, Jan 2005 - Aug 2008	Pulmonary units in university hospitals, cross-sectional	Stable COPD^§^	322/584 (55%)	Missing/incomplete data	Significant difference in sex distribution
Burgel et al. (2012), France, Jan 2005 - June 2009	Pulmonary units in university hospitals, prospective cohort	Stable COPD^§^	303/584 (52%)	Missing/incomplete data	Significant difference in sex distribution
Burgel et al. (2012), Belgium, Outcome assessed in Jan 2010	2 cohorts - Leuven university hospital COPD outpatient clinic, and from the NELSON study: community-based randomized lung cancer screening study, prospective cohort	• Smoking history ≥ 15 pack-years and age > 50 years (for the NELSON study) COPD (for the hospital cohort)	527/649 (81%) - 374/495 from COPD clinic, 153/154 from NELSON study	Missing/incomplete data	Significant difference in sex, age, FEV_1_% predicted, BMI and follow-up time
Cho et al. (2010), USA	17 university-based clinics, cross-sectional	• Self-identified white subjects Physician-diagnosed COPD FEV_1_ ≤ 45% predicted Hyperinflation on PFT Bilateral emphysema on CT scan	308/1220 (28%)	Missing/incomplete data	Significant difference in lung function parameters, 6MWD, PaCO_2_
DiSantostefano et al. (2013), USA, Canada 2004-2005	Data pooled from two studies conducted across 98, and 94 research sites, respectively (setting not specified), randomized controlled trials	• ≥40 years Clinical history of COPD Pre-bronchodilator FEV_1_ ≤ 50% predicted, FEV_1_/ FVC ratio ≤ 0.7 Smoking history ≥10 pack-years Documented history of ≥1 moderate/severe COPD exacerbations in the previous year	1543/1579 (98%)	Protocol violations	Not reported
Garcia-Aymerich et al. (2011), Spain, Jan 2004 - March 2006	9 teaching hospitals, prospective cohort	• Patients hospitalized for the first time with a COPD exacerbation COPD, diagnosed 3 months after discharge, when clinically stable	342/604 (57%)	Non-participation (213 patients refused, 23 patients discharged before the interview, 12 deaths, 14 lost to follow-up)	Significant difference in smoking status and a diagnosis of congestive heart failure
Spinaci et al. (1985), Italy, 1979-1980	University out-patient clinic, cross-sectional	Stable, severe COLD: FEV_1_ < 1.5 L and (ratio between FEV_1_/FVC <0.6	532	Not reported	Not reported
Vanfleteren et al. (2013), Netherlands, Nov 2007- Nov 2010	Tertiary care referral center for pulmonary rehabilitation program, cross-sectional	• Moderate to very severe COPD (GOLD stages II–IV) 40–80 years Clinically stable	213	Not reported	Not reported

^§^COPD defined by a post-bronchodilator FEV_1_/FVC ratio <0.7.

BMI – body mass index, 6MWD – six minute walk distance, PaCO_2_- Partial pressure of carbon dioxide in arterial blood, FEV_1_ – forced expiratory volume, 1 second, FVC-forced vital capacity, PFT – pulmonary function test.

### Studies reporting derivation of phenotypes for COPD that were excluded

We excluded 8 studies that involved the derivation of phenotypes for COPD. Five studies were excluded as none of the studies analyzed the association of the derived phenotypes with any clinically meaningful outcomes. The two studies by Paoletti et al. [16] and Pistolesi et al. [17] derived a set of 9 variables to predict airways obstructive or parenchymal destructive phenotypes based on cluster analysis used on computerized tomography (CT) parameters. The study by Camiciottoli et al. used principal component analysis to derive 2 variables that represented the phenotype (airway obstruction versus parenchymal destruction) and severity of the disease using CT scans, and then used multivariable regression to derive a set of variables to predict the two PCA-transformed variables [18]. Roy et al. [19] performed principal component analysis to derive four components to explain the variability in the dataset of 127 COPD patients. A recent study by Fens et al. [20] used factor analysis followed by k-means cluster analysis to derive four phenotypes for COPD using clinical, CT scan and breathomics-derived variables. We also excluded 3 studies [21-23] as they included subjects with both asthma and COPD, and in addition, did not report an association with clinical outcomes.

### COPD phenotyping in the selected studies

There was heterogeneity in the patient characteristics across studies. One study only included patients with a post-bronchodilator FEV_1_ ≤ 45% predicted [11], one study included patients with a pre-bronchodilator FEV_1_ ≤ 45% predicted [12] and one study only included subjects with GOLD 2-4 [15]. GOLD 1 subjects were under-represented in the studies that reported the classification of subjects by GOLD [8-10]. Women were under-represented across studies too; the proportion of women ranged from 7% [13] to 46% [12]. Table 2 summarizes the characteristics of the subjects in the selected studies for the systematic review; a more detailed description can be found in the online supplement, Additional file 1: Table S2.

**Table 2:** **Characteristics of subjects in the included studies of clinical phenotypes for COPD**

**Study**	**Classification of patients based on GOLD stage, n (%)**	**FEV**_**1**_ **(% predicted), mean (SD)** ^**§**^	**Age, mean (SD)** ^**§**^	**Proportion males (%)** ^**§**^	**Smoking exposure, pack years, mean (SD)** ^**§**^	**MMRC score, mean (SD)** ^**§**^
Burgel et al. (2010)	GOLD 1 - 21 (6.5) GOLD 2 -135 (42) GOLD 3 -107 (33) GOLD 4 - 59 (18)	GOLD 1 - 85 (82-86) GOLD 2 - 65 (59-71) GOLD 3 - 40 (34-45) GOLD 4 - 25 (21-29)	GOLD 1 - 66 (58-75) GOLD 2 - 66 (58-72) GOLD 3 - 64 (57-73) GOLD 4 - 63 (58-72)	GOLD 1 - 71 GOLD 2 - 79 GOLD 3 - 71 GOLD 4 - 85	GOLD 1 - 41 (28-56) GOLD 2 - 42 (26-55) GOLD 3 - 38 (25-50) GOLD 4 - 44 (30-72)	GOLD 1 - 1 (0-1) GOLD 2 - 1 (1-2) GOLD 3 - 2 (1-3) GOLD 4 - 3 (2-4)
Burgel et al. (2012)	Same as above	Same as above	Same as above	Same as above	Same as above	Same as above
Burgel et al. (2012)	GOLD 1 - 120 (22.8) GOLD 2 -169 (32.1) GOLD 3 -149 (28.3) GOLD 4 - 89 (16.9)	GOLD 1 - 93 (87-103) GOLD 2 - 64 (57-71) GOLD 3 - 40 (36-44) GOLD 4 - 24 (20-28)	GOLD 1 - 62 (58-67) GOLD 2 - 68 (61-74) GOLD 3 - 68 (62-75) GOLD 4 - 61 (58-65)	GOLD 1 - 80 GOLD 2 - 79 GOLD 3 - 78 GOLD 4 - 72	GOLD 1 - 43 (32-55) GOLD 2 - 47 (34-61) GOLD 3 - 50 (32-64) GOLD 4 - 46 (33-60)	GOLD 1 - 0 (0-1) GOLD 2 - 1 (0-2) GOLD 3 - 2 (1-3) GOLD 4 - 3 (1-3)
Cho et al. (2010)	Not reported. Only subjects with GOLD stages 3 and 4 included	28.3 (7.33)	67.4 (6.08)	197 (64)	67.4 (30.4)	Not reported
DiSantostefano et al. (2013)	Not reported. Only subjects with pre-bronchodilator FEV1 ≤ 50% predicted included	33.6 (25 – 41.9)	65 (59-72)	833 (54)	52 (40-77), 633 (41%) current smokers	Not reported
Garcia-Aymerich et al. (2011)	Not reported	52.4 (16.2)	67.9 (8.6)	318 (93)	Not reported, 109 (32.9%) current smokers	2 (2-3)
Spinaci et al. (1985)	Not reported, all subjects had a FEV_1_/VC < 0.6	FEV_1_/VC 41.06 (0.39)	59.5 (9.8)	398 (75)	Not reported, 274 (51.5%) current smokers	Not reported
Vanfleteren et al. (2013)	Not reported. Only subjects with GOLD stages 2 – 4 included	51.2 (16.9)	63.6 (7)	59	46 (26)	2.1 (1.09)

^§^The studies by Burgel et al. and the study by DiSantostefano et al. report median (inter-quartile range).

MMRC score – modified medical research council score for assessing degree of shortness of breath, SD – standard deviation.

There was significant heterogeneity in the methods used for cluster analysis, these have been summarized in Table 3, and a detailed summary of the variables included in each analysis is reported in the online supplement, Additional file 1: Table S3. None of the studies validated the derived phenotypes in an external cohort for clinically meaningful outcomes. Three studies prospectively validated the phenotypes with mortality data of the cohort from which the phenotypes were derived [9,10,13], and one of these also analyzed hospital admissions [13]. One of the studies [12] pooled the data from two randomized-controlled trials [24,25] to analyze the change in the frequency of exacerbations of individuals who were randomized to receive salmeterol/fluticasone propionate (SFC) compared to those who received salmeterol alone (SAL), and whether this response varied based on the phenotypes that were derived. The other studies validated the derived phenotypes with predicted mortality scores. These have been summarized in Table 3.

**Table 3:** **Statistical methods used for cluster analysis and outcomes tested in the included studies of clinical phenotypes for COPD**

**Study**	**Number of variables selected for analysis, method of selecting variables**	**Statistical method used for identifying phenotypes**	**Outcome tested**
Burgel et al. (2010)	8 variables, expert opinion	Cluster using k-means after variable reduction using PCA	BOD^§^ index
Burgel et al. (2012)	8 variables, expert opinion	Cluster using k-means after variable reduction using PCA	Mortality rates after 3.35 years of follow-up
Burgel et al. (2012)	18 variables, expert opinion	Hierarchical clustering with Wards method after variables reduction using PCA and MCA	Mortality rates after 17.2 months of follow-up
Cho et al. (2010)	43 variables (including 12 SNPs from 5 genes), expert opinion, and selection of genes included in previous genetic association studies	Cluster using k-means after variable reduction using factor analysis	Exacerbations/year over 3.3 years (retrospective)
DiSantostefano et al. (2013)	36 variables analyzed, co-linear variables dropped, expert opinion	Tree-based supervised cluster analysis using modified recursive partitioning	Decreased annual rate of exacerbations with SFC compared to SAL
Garcia-Aymerich et al. (2011)	224 variables, all variables collected (after excluding those with additive relationships or resulting from categorizations)	Cluster using k-means clustering method (PKM)	Admissions and mortality rates during a 4-year follow-up
Spinaci et al. (1985)	4 variables, expert opinion	Cluster using k -means clustering method (PKM)	Analysis of contingency tables
Vanfleteren et al. (2013)	13 variables, based on “clinical relevance and methodological possibilities to objectify the comorbidities”	Self- organizing maps (SOMs, or Kohonen maps) used to order patients by their overall similarity with regards to comorbidities. Clusters generated using a hybrid algorithm that applied classical hierarchical method of Ward on top of the SOM topology.	Updated BODE^¶^ index, Framingham 10-year risk

^§^BOD index –Body mass index (BMI), obstruction (FEV1% pred) and dyspnoea evaluated on the modified Medical Research Council (MMRC) scale). Celli B, Jones P, Vestbo J, et al. The multidimensional BOD:association with mortality in the TORCH trial. *Eur Respir J 2008; 32: Suppl. 52, 42 s.*

^¶^BODE index - Body mass index (BMI), obstruction (FEV1% pred), dyspnoea evaluated on the modified Medical Research Council (MMRC) scale), and exercise capacity on the 6-minute walk test. Celli B, et al. The Body-Mass Index, Airflow Obstruction, Dyspnea, and Exercise Capacity Index in Chronic Obstructive Pulmonary Disease. *N Engl J Med 2004; 350:1005-1012.*

PCA – Principal component analysis, MCA – Multiple component analysis.

Table 4 provides a summary of the derived phenotypes and their associations with studied outcomes. A more detailed description can be found in the online supplement, Additional file 1: Table S4. The number of phenotypes ranged from 2 to 5. Five of the studies described a phenotype of younger individuals with severe respiratory disease with a low prevalence of cardiovascular co-morbidities, high prevalence of poor nutritional status and poor health status [8-11,15]. In two of these studies, women were significantly over-represented in this phenotype [10,15]. A similar phenotype was reported in 2 other studies [13,14], but neither of the studies reported these patients to be younger than the rest of the cohort. Subjects with this phenotype had poor outcomes across the studies. All studies reported a phenotype of older individuals with moderate respiratory disease, and a high prevalence of obesity. In addition, 6 studies reported subjects with this phenotype to have an increased prevalence of cardiovascular and metabolic co-morbidities and inflammatory markers [8-10,12,13,15]. Subjects with this phenotype were reported to have a worse prognosis that the individuals with comparable age and respiratory disease status, and, in the study by DiSantostefano et al [12], they were found to have an improvement of prognosis with respect to a decrease in the frequency of exacerbations when treated with SFC, rather than SAL alone.

**Table 4:** **Description of the derived phenotypes and association with outcomes analyzed in the included studies of clinical phenotypes for COPD**

**Study**		**Phenotype 1**	**Phenotype 2**	**Phenotype 3**	**Phenotype 4**	**Phenotype 5**
Burgel et al. (2010)	Phenotype	• Young individual Very severe respiratory disease Frequent exacerbator Poor nutritional status Low prevalence of cardiovascular comorbidities High prevalence of depression and very poor HRQoL	• Older individual Mild respiratory disease High prevalence of overweight Low prevalence of cardiovascular comorbidities and depression Mildly impaired HRQoL	• Young individual Moderate respiratory disease Normal nutritional status Low prevalence of cardiovascular comorbidities and depression Moderately impaired HRQoL	• Older individual Moderate respiratory disease Frequent exacerbator High prevalence of overweight High prevalence of cardiovascular comorbidities and depression Poor HRQoL	
	N (%)	44 (13.7)	89 (27.6)	93 (28.9)	96 (29.8)	
	Outcome analyzed- BOD score*	5 (4-6)	1 (1-2)	3 (2-3)	4 (3-6)	
Burgel et al.^§^ (2012)	Outcome analyzed- crude mortality rate	15 (35%)	7 (8%)	17 (20%)	21 (25%)	
	Outcome analyzed- age at death, median, IQR	62 (58-68)	77 (66-83)	67 (58-69)	76 (74-79)	
	Age-adjusted mortality risk (Cox model)	8.35 (3.13,22.22) *v* Phenotype 2 1.91 (0.94,3.06) *v* Phenotype 3 3.18 (1.37,7.4) *v* Phenotype 4	Reference group with lowest mortality risk	4.33 (1.73,11.06) *v* Phenotype 2 1.67 (0.78, 3.57) *v* Phenotype 4	2.63 (1, 6.25) *v* Phenotype 2	
Burgel et al. (2012)	Phenotype	• Young individual Mild to moderate airflow limitation Absent or mild emphysema Absent or mild dyspnea Normal nutritional status Limited comorbidities	• Young individual Over-representation of women in the group Severe airflow limitation Marked emphysema and hyperinflation Low BMI Severe dyspnea Impaired HRQoL Osteoporosis, muscle weakness highly prevalent Diabetes and cardiovascular comorbidities less prevalent.	• Older individual Mostly male Moderate to severe airflow limitation Less severe emphysema than subjects in Phenotype 2 Higher prevalence of bronchial thickening Higher prevalence of obesity, diabetes and cardiovascular comorbidities		
	N (%)	219 (41.5)	99 (18.8)	209 (39.7)		
	Outcome analyzed- crude mortality rate	1 (0.5%)	20 (20.6%)	29 (14.3%)		
	Age-adjusted mortality risk (Cox model)	Reference group with lowest mortality risk	47.5 (6.3,358.6) *v* Phenotype 1 3.3 (1.5,7.2) *v* Phenotype 3	14.3 (1.9,110.3) *v* Phenotype 1		
Cho et al. (2010)	Phenotype	• Emphysema predominant Lower BMI Fewer pack-years of smoking Higher TLC, lower DLCO Lower 6MWD and maximum work	• Milder severity, fewer symptoms of dyspnea Fewer exacerbations, despite being of slightly older age Bronchodilator responsive Higher BMI Greater FVC and DLCO Lower PaCO_2_ Higher 6MWD and maximum work,	• Less emphysema and lower wall thickness (similar to Phenotype 2) Lower FEV_1_, less bronchodilator responsiveness, more dyspnea compared to Phenotype 2 despite a relatively younger age	• Airway predominant, highest airway thickness Higher BMI Lower TLC Less severe emphysema Lower PaO_2_ and lower 6MWD	
	N (%)	66 (21.4)	102 (33.1)	88 (28.6)	52 (16.9)	
	Outcome analyzed- exacerbations (retrospectively over 3.3 years)	0.19	0	0.19	0.15	
DiSantostefano et al. (2013)	Phenotype	• Treated with diuretics Higher BMI Fewer current smokers Frequent moderate exacerbations Higher use of cardiac medications and psycholeptics	• Not treated with diuretics Lower FEV_1_ Highest FEV_1_ reversibility post-bronchodilator Fewer proportion of subjects on cardiac medications and psycholeptics	• Not treated with diuretics Higher proportion of current smokers Higher FEV_1_ Lowest FEV_1_ reversibility post-bronchodilator		
	N (%)	454 (29)	756 (49)	333 (22)		
	Outcome analyzed- response in the rate of exacerbations to SFC as compared to SAL	Reduction in the annual rate of moderate/severe exacerbations among patients randomized to SFC as compared with SAL alone (RR = 0.56, p < 0.001);	Reduction in the annual rate of moderate/severe exacerbations among patients randomized to SFC as compared with SAL alone (RR = 0.67, p < 0.001)	No change in the annual rate of moderate/severe exacerbations among patients randomized to SFC as compared with SAL alone (RR = 1, p not significant)		
Garcia-Aymerich et al. (2011)	Phenotype	• Severe respiratory disease Poor functional capacity Emphysematous Few comorbidities	• Milder respiratory disease Preserved BMI Few comorbidities	• Mild respiratory disease High BMI Higher prevalence of comorbidities and inflammatory markers		
	N (%)	126 (36.9)	125 (36.5)	91 (26.6)		
	Outcome analyzed- ATS/ERS severity stage adjusted - COPD admission risk	2.89 (1.59 - 5.25)	Reference group with lowest mortality risk	1.54 (0.91 - 2.63)		
	Outcome analyzed- ATS/ERS severity stage adjusted -Mortality	2.01 (0.72 - 5.62)	Reference group with lowest mortality risk	1.55 (0.67 - 3.58)		
Spinaci et al. (1985)	Phenotype	• Severe respiratory disease Heavy smokers Emphysematous Frequent hospitalizations	• Milder respiratory disease Preserved body weight Lower prevalence of emphysema Fewer hospitalizations			
	N (%)	189 (36)	343 (64)			
	Outcome analyzed- Analysis of contingency tables	Worse prognosis of life (details not provided)				
Vanfleteren et al. (2013)	Phenotype	• Younger individuals Fewer comorbidities Higher HRQoL	• Older individuals Higher prevalence of cardiovascular comorbidities Poor HRQoL	• Younger individuals Women over-represented in the group Higher prevalence of emphysema Higher prevalence of underweight, muscle wasting, osteoporosis	• Predominantly male High prevalence of obesity, hyperglycemia, dyslipidemia and atherosclerosis	• Younger individuals • Severe dyspnea • High prevalence of anxiety and depression, • Poor HRQoL
	N (%)	67 (31.4)	49 (23)	44 (21)	33 (15.5)	20 (9.4)
	Outcome analyzed- Updated BODE score**	2.4 (2.6)	3.4 (3.3)	3 (1.8)	2.6 (2.3)	3.1 (1.9)
	Outcome analyzed- Framingham 10-year risk, %	8.6 (6.6)	11.5 (6.6)	7.6 (6)	11.9 (7.3)	6.6 (4.5)

*BOD score – Score calculated using body mass index (BMI), obstruction (FEV1 % pred) and dyspnoea evaluated on the modified Medical Research Council (MMRC) scale).

^§^The study was a longitudinal analysis of outcomes for the same cohort enrolled in the study by Burgel et al. (2010)^9.^

**BODE score - Score calculated using body mass index (BMI), obstruction (FEV1 % pred), dyspnoea evaluated on the modified Medical Research Council (MMRC) scale), and exercise capacity on the 6-minute walk test.

HRQoL – health-related quality of life, IQR – inter-quartile range, BMI – body-mass index, TLC – total lung capacity, DLCO- diffusion capacity of the lung for carbon monoxide, 6MWD- six-minute walk distance, FVC – forced vital capacity, PaCO_2_- Partial pressure of carbon dioxide in arterial blood, SFC – salmeterol/fluticasone propionate SAL - salmeterol.

### Assessment of quality of studies

The 8 studies were in compliance with most of the items on the STROBE checklist. There were methodological limitations with some of the studies. Only one study reported a rationale for the sample size [13]. Two studies did not report numbers of individuals at each stage of the study, nor used a flow diagram [14,15]. Only two studies conducted sensitivity analyses: one tested the robustness of the data to using the lower limit of normal (LLN) instead of the fixed ratio for the diagnosis of COPD [8], and the other repeated the cluster analysis using an alternative method, and also analyzed the results in subsets of included participants [13]. Only two studies imputed missing values for variables, one using the median for continuous variables, and the most frequent value for categorical variables [12], and the other using multiple imputation using chained equations [13]. One study reported the validation of the phenotypes with contingency tables, but failed to report any specific details [14].

## Discussion

This systematic review of the literature for COPD phenotype studies using unsupervised (free of definitive pre-determined hypotheses) statistical analyses to derive clusters associated with clinically relevant outcomes yielded 8 studies with significant heterogeneity in the selection of subjects, statistical methods used and outcomes validated. Despite these differences, two clinical phenotypes were consistently found across most studies, and may have clinical implications.

One of the phenotypes that describe individual been younger with severe respiratory disease, having a low probability of cardiovascular co-morbidities, high prevalence of poor nutritional status and poor health status with poor longitudinal health outcomes may be important for two reasons. Firstly, although the derivation of phenotypes was cross-sectional, it is likely that such individuals experience a rapid decline in lung function, and therefore, recognizing this phenotype at a younger age and treating the disease aggressively along with measures to support smoking cessation could have important prognostic implications. Secondly, given the low prevalence of cardiovascular co-morbidities, it is likely that such individuals would be good candidates for lung transplantation. Longitudinal cohort studies that include younger patients early in the course of their disease, and follow them closely to understand the differential progression of disease would be vital to a better understanding of this phenotype. Two of the studies found a high proportion of women in the sub-group of individuals with this phenotype, and a stronger pattern might emerge if more women are included in such studies. This phenotype was also associated with a lower height in one study [13], and the authors hypothesized that an impaired in-utero and childhood lung growth may have been a contributing factor to the severity of disease. This association needs to be further validated.

The other phenotype of older individuals with moderate respiratory disease, and a high prevalence of obesity, and increased prevalence of cardiovascular and metabolic co-morbidities and inflammatory markers is important as such individuals appear to have worse health outcomes than individuals with comparable respiratory disease with fewer co-morbidities. Contrary to the hypothesis of a “chronic systemic inflammatory syndrome” [26] that proposes a systemic inflammation attributable to COPD, Garcia-Aymerich et al. [13] found that bronchial inflammatory markers were not associated with systemic inflammatory markers. This suggests that the worse health outcomes might be caused by the co-morbidities, and consequently, screening for, and optimally treating the co-morbidities might be associated with better health outcomes. The study by DiSantostefano et al. also found that patients with this phenotype had a decreased frequency of moderate/severe exacerbations when treated with SFC, when compared to those with the same phenotype randomized to treatment with SAL alone [12].

The study by Vanfleteren et al. [15] identified a phenotype with a very high prevalence of anxiety and depression. A third of such patients had a myocardial infarction, and as suggested by the authors, is consistent with reports on anxiety being the strongest predictor of mortality in COPD [27]. The study by DiSantostefano et al. also found that a high proportion of individuals in the group with cardiovascular comorbidities were on psycholeptic drugs [12,14]. This is important as it is well established that depression and anxiety adversely affect prognosis in COPD, conferring an increased risk of exacerbation and possibly death [28]. Conversely, COPD also increases the risk of developing depression. Future studies should therefore screen patients with COPD for psychiatric disorders for a better understanding of this phenotype.

The present GOLD classification of COPD acknowledges the complexity of the disease, and has included measures of risk, in addition to classification based on severity of the spirometric abnormality [3]. A comparison of the predictive ability of phenotypes and that of the present GOLD classification with regards to clinically relevant outcomes would help in our understanding of the disease process, and needs to be explored by future studies.

Several methodological limitations were observed in the studies, and the elaboration of these limitations will hopefully serve to guide future studies. In a biased sample, the derived phenotypes merely reflect the bias in selection, and not necessarily the heterogeneity of disease. Of the 7 studies that specified the setting, all [8,9,11,13-15] except a subset on one study [10] were conducted in university-based referral centers, and patients with mild, early disease. In most developed countries, the prevalence of COPD in women has been reported to be comparable to that of men in recent years [29] and it therefore appears that women were under-represented in most included studies. Four studies [8-11] reported missing data, and only subjects with complete data were analyzed. This lead to a significant proportion of patients being excluded; the excluded patients were found to be statistically significantly different from those who were included on several important parameters. Strategies such as multiple imputations, employed by two studies [12,13], may be of use in the design of future studies.

Studies used different statistical methodologies for the derivation of phenotypes. Three studies [8-10] used principal component analysis for reducing the dimensionality of the data; the clinical meaningfulness of principal components can be difficult to interpret. Cho et al. [11] used factor analysis, followed by several clustering algorithms, a method that is considered robust. However, the study had a sample that was biased toward very severe disease, and the external validity of the results needs to be tempered. DiSantostefano et al. used tree-based supervised cluster analysis using modified recursive partitioning to derive clusters. Self-organizing maps, used in one study [15], are a newer method that needs further validation. A summary of the strengths and weaknesses of the various clustering techniques can be found in a recent review by the authors of this study [30]. Only one study internally validated the clusters by splitting the dataset for derivation and validation [12], but none of the studies validated the derived phenotypes in an external population, limiting the validity of these derived phenotypes. Few studies investigated the robustness of the derived phenotypes to (i) statistical methods used to perform the clustering; (ii) variables used to define the clusters. The use of prognostic indicators such as the BODE index and contingency tables, employed by three of the studies [8,14,15], is far less robust than the use of clinical endpoints to validate the phenotypes, and this limits the validity of the results of these studies. Two of the studies included subjects enrolled in randomized controlled trials (RCTs) [11,12], and it is likely that the populations selected were less heterogeneous than those based in the community, and the studies were likely to suffer from selections biases inherent to RCTs. Lastly, as all of the studies derived phenotypes with cross-sectional data; the stability of these phenotypes over time, and the effect of medications and interventions remain unknown, and needs to be studied in prospective studies.

The strengths of this systematic review include an *a priori* protocol, detailed literature search with no language restrictions, performed by a librarian (TL), independent review by two reviewers at every stage of the review, and exhaustive data extraction from included studies. The involvement of biostatisticians (AB, TZ) with expertise in this research field, significantly helped our understanding of the literature.

A major limitation of the study is the exclusion of studies that included patients with asthma. This possibly explains the lack of the asthma-COPD overlap phenotype [31] in the included studies. However, the exclusion was per protocol, with the aim of having the systematic review focused to answering a specific question. We also excluded studies that tested specific hypothesis-driven, or empiric phenotypes without a derivation study, reflecting the specific research question that the review was aimed to answer.

## Conclusion

This systematic review of the literature identified studies in which two phenotypes of COPD were reported often, representing different aspects of the disease spectrum, and recognizing these phenotypes and treating them optimally may have implications for altering the course of the disease. However, the selection of specific subsets of patients, evident in the existing studies, limits the generalizability of the results. This systematic review has provided important information on limitations of the phenotype studies in COPD and the need for improvement in future research. There is a need for sampling different populations in the COPD disease spectrum, to mirror the population of COPD patients at large, including women, never smokers, and those with mild disease. There is also a need for longitudinal studies of patients with COPD to validate in the same cohort and to explore sources of variability in phenotypes and their temporal nature as well as to allow an iterative validation process in which candidate phenotypes are identified before their relevance to clinical outcome is determined.

## Appendix 1: COPD Phenotypes: search strategy (May 13^th^, 2013)

The following databases were searched for relevant studies: MEDLINE (via OvidSP 1946 to 22/Apr/2013; via PubMed 1946 to 22/Apr/2013) ; Embase Classic + Embase (via OvidSP 1947 to 19/Apr/2013); BIOSIS Previews (via OvidSP 1969 to 2013 Week 20); Web of Science (via ThomsonReuters 1996 to 22/Apr/2013); Scopus (via Elsevier 1996 to 22/Apr/2013); CENTRAL (via Cochrane Library). The search strategy used text words and relevant indexing to answer the following question: What are the different phenotypes of COPD, based on subject characteristics? The full MEDLINE strategy, shown below, was applied to all databases, with modifications to search terms as necessary.

1. exp Pulmonary Disease, Chronic Obstructive/ (20075)
2. Lung Diseases, Obstructive/ (17796)
3. exp Pulmonary Emphysema/ (13525)
4. (obstructive adj2 (pulmonary or lung$ or respirat$ or air$)).tw. (32407)
5. (chronic air$ adj2 (obstruction$ or limitation$ or occlusion$)).tw. (1332)
6. (chronic bronch$ adj2 (obstruction$ or limitation$ or occlusion$)).tw. (90)
7. (chronic$ adj2 bronch$).tw. (12047)
8. COPD.tw. (22440)
9. COAD.tw. (185)
10. emphysema$.tw. (19592)
11. or/1-10 (81067)
12. exp Phenotype/ (202484)
13. Genetics/ (11665)
14. exp Genotype/ (268840)
15. (phenotyp$ or genotyp$).tw. (481741)
16. (clusters or clustering).tw. (107696)
17. exp cluster analysis/ (38617)
18. (cluster$ adj3 analys$).tw. (17931)
19. or/12-18 (832022)
20. 11 and 19 (2920)
21. exp Tomography, X-Ray/ (284476)
22. Tomography/ (8768)
23. exp Diagnostic Techniques, Respiratory System/ (227864)
24. exp Biological Markers/ (579344)
25. “Quality of Life”/ (106896)
26. Sickness Impact Profile/ (5735)
27. exp “Severity of Illness Index”/ (154452)
28. Disease Progression/ (92654)
29. exp Mortality/ (262597)
30. mortality.fs. (387945)
31. tomograph$.tw. (228779)
32. cine ct.tw. (132)
33. (c?t adj2 (scan$ or ray$)).tw. (65050)
34. (tomodensitomet$ or tomo densitomet$).tw. (782)
35. (xray$ or “x ray$”).tw. (216447)
36. (bronchograph$ or bronchoscop$).tw. (20406)
37. (mucocil?iary adj2 (clearance$ or transport$)).tw. (2580)
38. ((blood or pulmonary or lung$) adj2 gas$2).tw. (22758)
39. plethysmograph$.tw. (11082)
40. ((respiratory or ventilation) adj2 air$).tw. (2842)
41. (ergomet$ adj2 test$).tw. (2380)
42. (test$ adj2 (exercis$ or tre?dmill or stress or step or stepping)).tw. (35120)
43. spiromet$.tw. (14294)
44. ((lung$ or respiratory or pulmonary or air$ or bronch$) adj2 (function$ or capacit$ or compliance$ or ventilat$)).tw. (74259)
45. ((lung$ or respiratory or pulmonary or air$ or bronch$ or ventilation) adj2 (test$ or assess$ or evaluat$ or measur$)).tw. (51207)
46. (breath$ adj2 work$).tw. (1554)
47. ((breath$ or respirat$ or lung$) adj2 sound$).tw. (1284)
48. (stridor$ or rale$ or crackle$ or ronchi$ or ronchus or wheezing$).tw. (10954)
49. (pleural adj2 rub$).tw. (31)
50. sputum.tw. (21066)
51. (qualit$ adj2 life).tw. (133643)
52. QOL.tw. (16886)
53. ((sickness or illness or disease) adj2 (impact$ or profile$ or severity)).tw. (35074)
54. SIP.tw. (1744)
55. (exacerbat$ or deteriorat$).tw. (135724)
56. (expiratory adj3 (volume$ or flow or rate$)).tw. (20622)
57. (“vital capacit$” adj2 timed).tw. (44)
58. (FEV or FEV1 or “FEV 1” or FEVT or “FEV T”).tw. (21424)
59. (modified adj2 “medical research council”).tw. (155)
60. MMRC.tw. (82)
61. (george$ adj2 “respiratory questionnaire$”).tw. (908)
62. SGRQ.tw. (648)
63. mortality.tw. (423721)
64. or/21-63 (2631848)
65. 20 and 64 (1589)
66. limit 65 to animals (171)
67. limit 66 to humans (98)
68. 66 not 67 (73)
69. 65 not 68 (1516)
70. limit 69 to “all child (0 to 18 years)” (172)
71. limit 70 to “all adult (19 plus years)” (122)
72. 70 not 71 (50)
73. 69 not 72 (1466)
74. remove duplicates from 73 (1422)

Proceedings of the American Thoracic Society (ATS), American College of Chest Physicians (ACCP) and the European Respiratory Society Congress (ERS) were searched from 2009-2012, 2003-2012 and 2006-2012, respectively.

## Additional file

Additional file 1
**Table S1:** Characteristics of the included studies. **Table S2:** Characteristics of subjects in the included studies. **Table S3:** Variables selected for analysis. **Table S4:** Characteristics of the derived phenotypes.

## References

[1] Chapman KR, Mannino DM, Soriano JB, Vermeire PA, Buist AS, Thun MJ (2006). Epidemiology and costs of chronic obstructive pulmonary disease. Eur Respir J.

[2] Halbert RJ, Natoli JL, Gano A, Badamgarav E, Buist AS, Mannino DM (2006). Global burden of COPD: systematic review and meta-analysis. Eur Respir J.

[3] Vestbo J, Hurd SS, Agusti AG, Jones PW, Vogelmeier C, Anzueto A (2013). Global strategy for the diagnosis, management, and prevention of chronic obstructive pulmonary disease: GOLD executive summary. Am J Respir Crit Care Med.

[4] Han MK, Agusti A, Calverley PM, Celli BR, Criner G, Curtis JL (2010). Chronic obstructive pulmonary disease phenotypes: the future of COPD. Am J Respir Crit Care Med.

[5] Vogt W, Nagel D (1992). Cluster analysis in diagnosis. Clin Chem.

[6] Liberati A, Altman DG, Tetzlaff J, Mulrow C, Gotzsche PC, Ioannidis JPA (2009). The PRISMA statement for reporting systematic reviews and meta-analyses of studies that evaluate health care interventions: Explanation and elaboration. Ann Intern Med.

[7] von Elm E, Altman DG, Egger M, Pocock SJ, Gotzsche PC, Vandenbroucke JP (2007). The strengthening the reporting of observational studies in epidemiology (STROBE) statement: guidelines for reporting observational studies. Lancet.

[8] Burgel PR, Paillasseur JL, Caillaud D, Tillie-Leblond I, Chanez P, Escamilla R (2010). Clinical COPD phenotypes: a novel approach using principal component and cluster analyses. Eur Respir J.

[9] Burgel PR, Roche N, Paillasseur JL, Tillie-Leblond I, Chanez P, Escamilla R (2012). Clinical COPD phenotypes identified by cluster analysis: validation with mortality. Eur Respir J.

[10] Burgel PR, Paillasseur JL, Peene B, Dusser D, Roche N, Coolen J (2012). Two distinct chronic obstructive pulmonary disease (COPD) phenotypes are associated with high risk of mortality. PLoS One.

[11] Cho MH, Washko GR, Hoffmann TJ, Criner GJ, Hoffman EA, Martinez FJ (2010). Cluster analysis in severe emphysema subjects using phenotype and genotype data: an exploratory investigation. Respir Res.

[12] Disantostefano RL, Li H, Rubin DB, Stempel DA (2013). Which patients with chronic obstructive pulmonary disease benefit from the addition of an inhaled corticosteroid to their bronchodilator? A cluster analysis. BMJ Open.

[13] Garcia-Aymerich J, Gomez FP, Benet M, Farrero E, Basagana X, Gayete A (2011). Identification and prospective validation of clinically relevant chronic obstructive pulmonary disease (COPD) subtypes. Thorax.

[14] Spinaci S, Bugiani M, Arossa W, Bucca C, Rolla G (1985). A multivariate analysis of the risk in chronic obstructive lung disease (COLD). J Chronic Dis.

[15] Vanfleteren LE, Spruit MA, Groenen M, Gaffron S, van Empel VP, Bruijnzeel PL (2013). Clusters of comorbidities based on validated objective measurements and systemic inflammation in patients with chronic obstructive pulmonary disease. Am J Respir Crit Care Med.

[16] Paoletti M, Camiciottoli G, Meoni E, Bigazzi F, Cestelli L, Pistolesi M (2009). Explorative data analysis techniques and unsupervised clustering methods to support clinical assessment of Chronic Obstructive Pulmonary Disease (COPD) phenotypes. J Biomed Inform.

[17] Pistolesi M, Camiciottoli G, Paoletti M, Marmai C, Lavorini F, Meoni E (2008). Identification of a predominant COPD phenotype in clinical practice. Respir Med.

[18] Camiciottoli G, Bigazzi F, Paoletti M, Cestelli L, Lavorini F, Pistolesi M (2013). Pulmonary function and sputum characteristics predict CT phenotype and severity of COPD. Eur Respir J.

[19] Roy K, Smith J, Kolsum U, Borrill Z, Vestbo J, Singh D (2009). COPD phenotype description using principal components analysis. Respir Res.

[20] Fens N, van Rossum AG, Zanen P, van Ginneken B, van Klaveren RJ, Zwinderman AH (2013). Subphenotypes of mild-to-moderate COPD by factor and cluster analysis of pulmonary function, CT imaging and breathomics in a population-based survey. Copd.

[21] Jo KW, Ra SW, Chae EJ, Seo JB, Kim NK, Lee JH (2010). Three phenotypes of obstructive lung disease in the elderly. Int J Tuberc Lung Dis.

[22] Marsh SE, Travers J, Weatherall M, Williams MV, Aldington S, Shirtcliffe PM (2008). Proportional classifications of COPD phenotypes. Thorax.

[23] Weatherall M, Travers J, Shirtcliffe PM, Marsh SE, Williams MV, Nowitz MR (2009). Distinct clinical phenotypes of airways disease defined by cluster analysis. Eur Respir J.

[24] Anzueto A, Ferguson GT, Feldman G, Chinsky K, Seibert A, Emmett A (2009). Effect of fluticasone propionate/salmeterol (250/50) on COPD exacerbations and impact on patient outcomes. Copd.

[25] Ferguson GT, Anzueto A, Fei R, Emmett A, Knobil K, Kalberg C (2008). Effect of fluticasone propionate/salmeterol (250/50 microg) or salmeterol (50 microg) on COPD exacerbations. Respir Med.

[26] Fabbri LM, Rabe KF (2007). From COPD to chronic systemic inflammatory syndrome?. Lancet.

[27] Divo M, Cote C, de Torres JP, Casanova C, Marin JM, Pinto-Plata V (2012). Comorbidities and risk of mortality in patients with chronic obstructive pulmonary disease. Am J Respir Crit Care Med.

[28] Atlantis E, Fahey P, Cochrane B, Smith S (2013). Bidirectional associations between clinically relevant depression or anxiety and COPD: a systematic review and meta-analysis. Chest.

[29] Aryal S, Diaz-Guzman E, Mannino DM (2013). COPD and gender differences: an update. Transl Res.

[30] Bourbeau J, Pinto LM, Benedetti A (2014). Phenotyping of COPD: challenges and next steps. Lancet Respiratory Med.

[31] Gibson PG, Simpson JL (2009). The overlap syndrome of asthma and COPD: what are its features and how important is it?. Thorax.

