# Supplementary material for: Derivation and validation of clinical phenotypes for COPD: a systematic review
Source: Respir Res. 2015 Apr 18;16(1):50. doi: 10.1186/s12931-015-0208-4 (PMC4460884; doi:10.1186/s12931-015-0208-4)
Supplement: Additional file 1 — Table S1: Characteristics of the included studies. Table S2: Characteristics of subjects in the included studies. Table S3: Variables selected for analysis. Table S4: Characteristics of the derived phenotypes. [file 12931_2015_208_MOESM1_ESM.doc]

**Table S1 Characteristics of the included studies**

| **Study, Country** | **Study period** | **Setting** | **Design** | **Inclusion criteria** | **Exclusion criteria** | **Number enrolled** | **Number included in the analysis** | **Reason for exclusion** | **Difference between included and excluded patients** |
| --- | --- | --- | --- | --- | --- | --- | --- | --- | --- |
| Burgel et al. (2010)^9^, France | Jan 2005 - Aug 2008 | Pulmonary units in university hospitals | Cross-sectional | • Post- bronchodilator FEV_1_/forced vital capacity (FVC) ratio < 70%  • Stable condition (no history of exacerbation requiring medical treatment for the previous 4 weeks) | • Bronchiectasis • Asthma or  • Any significant respiratory diseases | 584 | 322 | Missing data | Significant difference in sex distribution |
| Burgel et al. (2012) ^10^, France | Jan 2005 - June 2009 | Pulmonary units in university hospitals | Prospective cohort | • Post- bronchodilator FEV_1_/forced vital capacity (FVC) ratio < 70%  • Stable condition (no history of exacerbation requiring medical treatment for the previous 4 weeks) | • Bronchiectasis • Asthma or  • Any significant respiratory diseases | 584 | 303 | Missing data | Significant difference in sex distribution |
| Burgel et al. (2012) ^11^, Belgium | Outcome assessed in Jan 2010 | 2 cohorts: 1. Leuven university hospital COPD outpatient clinic 2.NELSON study: community-based randomized lung cancer screening study | Prospective cohort | • Smoking history ≥ 15 pack-years and age > 50 years (for the NELSON study) • Post- bronchodilator FEV_1_/forced vital capacity (FVC) ratio < 70% | Not reported | 649 ; 495 from COPD clinic, 154 from NELSON study | 527; 374 from COPD clinic, 153 from NELSON study | Missing data | Significant difference in sex, age, FEV_1_ % predicted, BMI and follow-up time |
| Cho et al. (2010) ^12^, USA |  | 17 university-based clinics | Cross-sectional | • Self-identified white subjects  • Physician-diagnosed COPD  • FEV_1_ ≤ 45% predicted • Evidence of hyperinflation on pulmonary function testing • Bilateral emphysema on chest CT scan | • Postrehabilitation, postbronchodilator FEV_1_ ≤20% predicted and either non- heterogeneous emphysema on CT scan or DLCO ≤20% predicted • CT scan evidence of diffuse emphysema judged unsuitable for LVRS • Clinically significant bronchiectasis • Myocardial infarction within 6 months of interview and ejection fraction <45% • Congestive heart failure within 6 months of interview and ejection fraction <45% • History of recurrent infections with daily sputum production judged clinically significant • Six-minute walk distance ≤140 m postrehabilitation | 1120 | 308 | Incomplete CT phenotypic data | Significant difference in lung function parameters, 6MWD, PaCO_2_ |
| DiSantostefano et al. (2013) ^13^, USA | 2004-2005 | Data pooled from two studies conducted across 98, and 94 research sites, respectively (setting not specified) | Randomized controlled trials | - ≥ 40 years - Clinical history of COPD - Pre-bronchodilator FEV1 ≤50% predicted, FEV1/ FVC ratio ≤ 0.7 - Smoking history ≥10 pack-years - Documented history of ≥1 moderate/severe COPD exacerbations in the previous year | - Current diagnosis of asthma based on the American Thoracic Society standards for diagnosis - Other active chronic respiratory disorders apart from COPD - A moderate/severe exacerbation that had not resolved prior to visit 1 - Concurrent use of anticholinergics, theophyllines and leukotriene modifiers - History or current significant health conditions that could affect subject safety or effectiveness evaluation if the condition exacerbated during the study, such as clinically significant cardiac arrhythmias, uncontrolled/unstable congestive heart failure, uncontrolled hypertension or unstable angina | 1543 | 1579 | Protocol violations | Not reported |
| Garcia-Aymerich et al. (2011) ^14^, Spain | Jan 2004 - March 2006 | 9 teaching hospitals, | Prospective cohort | • Patients hospitalized for the first time with a COPD exacerbation  • Post- bronchodilator FEV_1_/FVC ratio < 70%, 3 months after discharge, when clinically stable | • Patients under 45 years of age  • Severe comorbidity, i.e. tuberculosis with residual lesion affecting more than 1/3 of parenchyma, pneumectomy or diagnosed pneumoconiosis, advanced cancer, psychiatric disorder, severe cardiovascular or neurological disease, and other • mental incapacity •frail or elderly patients with any disability that would hinder participation in the study  • Not living in the healthcare area of that particular hospital  • Not understanding the language. | 604 | 342 | Non-participation (213 patients refused, 23 patients discharged before the interview, 12 deaths, 14 lost to follow-up) | Significant difference in smoking status and a diagnosis of congestive heart failure |
| Spinaci et al. (1985) ^15^, Italy | 1979-1980 | University out-patient clinic | Cross-sectional | - Severe COLD: FEVI < 1.5 L and (ratio between FEV1 and vital capacity<60 % & confirmed in 2 consecutive observations in the last 3 months - Stable clinical condition | - Fibrotic or infiltrative lung diseases - Pneumoconiosis - Kyphoscoliosis - Other life threatening illnesses. | Not reported | 532 |  |  |
| Vanfleteren et al. (2013) ^16^, Netherlands | Nov 2007- Nov 2010 | Tertiary care referral center for pulmonary rehabilitation program | Cross-sectional | - Moderate to very severe COPD (GOLD grades II–IV) - 40–80 years - Clinically stable state | - History of asthma - α_1_-antitrypsin deficiency - Previous lung surgery - Active inflammatory disease - Acute myocardial infarction within the last 6 months - Known bone disease other than osteoporosis - Current or recently (<5 yr before the study) treated malignant disease - Use of high-dose systemic glucocorticosteroids (>10 mg prednisolone) | 213 | 213 |  |  |

**Table S2 Characteristics of subjects in the included studies**

| **Study** | **BMI** | **Exacerbations per patient per year** | **Coronary artery disease, %** | **Chronic heart failure, %** | **Diabetes** | **Hypertension** |
| --- | --- | --- | --- | --- | --- | --- |
| Burgel et al. (2010) ^9^ | GOLD 1 - 27 (24-28) GOLD 2 - 25 (23-29) GOLD 3 - 24 (20-27) GOLD 4 - 22 (18-26) | GOLD 1 - 2 (0-3) GOLD 2 - 1 (0-2) GOLD 3 - 2 (1-3) GOLD 4 - 2 (1-5) | GOLD 1 - 19 GOLD 2 - 20 GOLD 3 - 18 GOLD 4 - 5 | GOLD 1 - 9.5 GOLD 2 - 17.7 GOLD 3 - 18.3 GOLD 4 - 19.3 | GOLD 1 - 10 GOLD 2 - 5 GOLD 3 - 11 GOLD 4 - 11 | GOLD 1 - 48 GOLD 2 - 44 GOLD 3 - 36 GOLD 4 - 23 |
| Burgel et al. (2012) ^10^ | Same as above | Same as above | Same as above | Same as above | Same as above | Same as above |
| Burgel et al. (2012) ^11^ | GOLD 1 - 25 (24-28) GOLD 2 - 26 (23-28) GOLD 3 - 24 (20-27) GOLD 4 - 22 (19-25) | Not reported | GOLD 1 - 14 GOLD 2 - 27 GOLD 3 - 23 GOLD 4 - 26 | Not reported | GOLD 1 - 8 GOLD 2 - 17 GOLD 3 - 14 GOLD 4 - 13 | Not reported |
| Cho et al. (2010) ^12^ | 25.1 (3.45) | 0.16 (0-3.12) | Not reported | Not reported | Not reported | Not reported |
| DiSantostefano et al. (2013) ^13^ | 27 (23-31) | 63% had one exacerbation, 22% had 2 exacerbations/yr, 15% had ≥ 3 exacerbations/yr | Not reported | 29% were being treated with diuretics | 11% | 4% were being treated with anti-hypertensives |
| Garcia-Aymerich et al. (2011) ^14^ | 28.2 | Not reported | 11 | 7 | 19 | Not reported |
| Spinaci et al. (1985) ^15^ | Not reported | Not reported | Not reported | Not reported | Not reported | Not reported |
| Vanfleteren et al. (2013) ^16^ | 26.2 (5.1) | Not reported | 9% Myocardial infarction, 53% atherosclerosis | 2.8 | 5.6 | 48 |

**Table S2 (continued) Characteristics of subjects in the included studies**

| **Study** | **HAD scale total** | **HAD scale anxiety** | **HAD scale depression** | **SGRQ total score** | **Clinical COPD questionnaire, total score** |
| --- | --- | --- | --- | --- | --- |
| Burgel et al. (2010) ^9^ | GOLD 1 - 15 (8-19) GOLD 2 - 13 (9-17) GOLD 3 - 13 (8-18) GOLD 4 - 14 (8-21) | GOLD 1 - 8 (5-11) GOLD 2 - 7 (5-10) GOLD 3 - 7 (5-10) GOLD 4 - 8 (4-11) | GOLD 1 - 6 (3-10) GOLD 2 - 6 (3-8) GOLD 3 - 5 (3-9) GOLD 4 - 7 (3-11) | GOLD 1 - 27 (16-57) GOLD 2 - 36 (27-52) GOLD 3 - 51 (38-61) GOLD 4 - 64 (46-72) | Not reported |
| Burgel et al. (2012) ^10^ | Same as above | Same as above | Same as above | Same as above | Not reported |
| Burgel et al. (2012) ^11^ | Not reported | Not reported | Not reported | Not reported | GOLD 1 - 1.8 (0.8-3.5) GOLD 2 - 3.5 (1.8 - 6.3) GOLD 3 - 5.5 (3.5-7.8) GOLD 4 - 6.8 (5.3-9) |
| Cho et al. (2010) ^12^ | Not reported | Not reported | Not reported | Not reported | Not reported |
| DiSantostefano et al. (2013) ^13^ | Not reported | Not reported | Not reported | Not reported | Not reported |
| Garcia-Aymerich et al. (2011) ^14^ | Not reported | Not reported | Not reported | Not reported | Not reported |
| Spinaci et al. (1985) ^15^ | Not reported | Not reported | Not reported | Not reported | Not reported |
| Vanfleteren et al. (2013) ^16^ | 16% had scores that were above the cut-off for depression | 21% had scores that were above the cut-off for anxiety | Not reported | 51.3 (17.5) | Not reported |

**Table S2 (continued) Characteristics of subjects in the included studies**

| Study | CT-detected emphysema present, % | CT-detected Mild/moderate/severe bronchial thickening, % | CT-detected bronchiectasis, % | LABA use, % | ICS use, % | Tiotropium use, % |
| --- | --- | --- | --- | --- | --- | --- |
| Burgel et al. (2010) ^9^ | Not reported | Not reported | Not reported | GOLD 1 - 52 GOLD 2 - 56 GOLD 3 - 74 GOLD 4 - 85 | GOLD 1 - 52 GOLD 2 - 55 GOLD 3 - 73 GOLD 4 - 78 | GOLD 1 - 19 GOLD 2 - 23 GOLD 3 - 18 GOLD 4 - 19 |
| Burgel et al. (2012) ^10^ | Not reported | Not reported | Not reported | Same as above | Same as above | Same as above |
| Burgel et al. (2012) ^11^ | GOLD 1 - 39 GOLD 2 - 69 GOLD 3 - 82 GOLD 4 - 92 | GOLD 1 - 64/30/6 GOLD 2 - 37/45/18 GOLD 3 - 24/49/27 GOLD 4 - 32/48/20 | GOLD 1 - 12 GOLD 2 - 26 GOLD 3 - 29 GOLD 4 - 32 | Not reported | Not reported | Not reported |
| Cho et al. (2010) ^12^ | Not reported | Not reported | Not reported | Not reported | Not reported | Not reported |
| DiSantostefano et al. (2013) ^13^ | Not reported | Not reported | Not reported | Not reported | Not reported | Not reported |
| Garcia-Aymerich et al. (2011) ^14^ | Not reported | Not reported | Not reported | Any respiratory drug treatment 288 (85.2%) | Not reported | Not reported |
| Spinaci et al. (1985) ^15^ | Not reported | Not reported | Not reported | Not reported | Not reported | Not reported |
| Vanfleteren et al. (2013) ^16^ | Not reported | Not reported | Not reported | 19 | 12 | 78 |

**Table S3 Variables selected for analysis**

| Study | Variables analyzed |
| --- | --- |
| Burgel et al. (2010) ^9^ | - Age (years) - Tobacco smoking (pack-years) - Severity of airflow obstruction (assessed by FEV_1_ % pred) - Exacerbations (number per patient per year) - Nutritional status (assessed by BMI) - Dyspnoea (assessed by the MMRC scale) - HRQoL (assessed by the SGRQ total score) - Anxiety and depression (assessed by the HAD total score) |
| Burgel et al. (2012) ^10^ | Same as above |
| Burgel et al. (2012) ^11^ | - Age (years) - Nutritional status (assessed by BMI) - Severity of airflow obstruction (assessed by FEV_1_ % pred) - Dyspnoea (assessed by the MMRC scale) - HRQoL (assessed by the CCQ total score) - Presence of hyperinflation (assessed by thoracic gas volume, TGV, % predicted) - Alveolar destruction (assessed by diffusing capacity, DL_CO_, % predicted) - All categorical variables including comorbidities, and data obtained from CT analysis, including emphysema, bronchial thickening and bronchiectasis. |
| Cho et al. (2010) ^12^ | - Age, years - BMI, kg/m2 - Gender (% male) - Pack-years of smoking - Age started smoking - Age quit smoking - Pre-bronchodilator FEV_1_, % predicted - Pre-bronchodilator FVC, % predicted - Post-bronchodilator FEV_1_, % predicted - Post-bronchodilator FVC, % predicted - Bronchodilator response, % of baseline FEV_1_ - Bronchodilator response, absolute change in FEV_1_, L - FEV_1_/FVC ratio, post-bronchodilator - FEV_1_/FVC ratio, pre-bronchodilator - Total lung capacity, % predicted - Residual volume, % predicted - Diffusion capacity, % predicted - Total fraction emphysema at -950 HU - Difference between apical and basal emphysema at -950 HU - Apical fraction emphysema at -950 HU - Airway wall thickness, mm - Airway wall area, % - Square root wall area, cm - 6 minute walk distance, ft - Maximum work, watts - UCSD Shortness of Breath Questionnaire - Arterial pH - PaO_2_, mmHg - PaCO_2_, mmHg - Exacerbations in year prior to randomization - Exacerbations/year (over 3.3 years).   Genes included: EPHX1, GSTP1, SERPINE2, SFTPB, TGFB1 |
| DiSantostefano et al. (2013) ^13^ | - Age (years) - Gender - Smoking status (current/former) - Pack-years of smoking - Body mass index - FEV_1_% predicted - FEV_1_% reversibility - FEV_1_/FVC ratio postalbuterol - FVC % predicted - SGRQ Activity score - SGRQ Impact score - SGRQ Symptom score - SGRQ Total score - Duration of COPD (years) - Chronic bronchitis (self-reported, yes/no) - Emphysema (self-reported, yes/no) - Exacerbations requiring hospitalisation (past 12 months) - Exacerbations requiring OCS/ antibiotics (past 12 months) - Gold Stage indicator variables based on lung function (II, III/IV) - Agents acting on the renin- angiotensin system (angiotensin converting enzyme inhibitors) - Anti-anaemic preparations - Anti-haemorrhagics - Anti-histamines - Anti-hypertensives - Anti-thrombotics - Anti-inflammatory and antirheumatic products - β-blockers - Bone disease (including muscle pain) medications - Calcium channel blockers - Cardiac therapies - Diabetes medications - Diuretics - Lipid-modifying agents - Psychoanaleptics - Psycholeptics - Vasodilators |
| Garcia-Aymerich et al. (2011) ^14^ | - Dyspnoea (mMRC scale) - SGRQ -Activity - Pre-bronchodilator and post-bronchodilator FEV_1_ (% predicted) - TGV; % predicted - IC/TLC ratio - PaO2 - Peripheral blood neutrophil count - Body weight - BMI |
| Spinaci et al. (1985) ^15^ | Variables such as (detailed list not provided): FEV_1_, VC, PaO_2_, PaCO_2_, HbO_2_, heart rate (HR) and change in FEV_1_. |
| Vanfleteren et al. (2013) ^16^ | - Chronic kidney disease (estimated glomerular filtration rate , 60 ml/min) - Anemia (hemoglobin level < 8.1 mmol/L in men and < 7.5 mmol/L in women) - Hypertension (systolic blood pressure ≥140 mm Hg or diastolic pressure ≥90 mm Hg) - Obesity (BMI >30 kg/m^2^) - Underweight (BMI < 21 kg/m2) - Muscle wasting (fat free mass index < 16 kg/m2 for men or <15 kg/m2 for women) - Hyperglycemia (fasting glucose level ≥ 5.6 mmol/L) - Dyslipidemia (triglyceride level ≥ 1.7 mmol/L or high-density lipoprotein cholesterol level ≥ 1.03 mmol/L in men or ≥ 1.29 mmol/L in women) - Osteoporosis (t-score less than 22.5) - Symptoms of anxiety and depression (HADS score >10 points) - Atherosclerosis (c-IMT ≥ 0.9 mm) - Myocardial infarction (CIIS >20) |

**Table S4 Characteristics of the derived phenotypes**

| **Study** |  | **Phenotype 1** | **Phenotype 2** | **Phenotype 3** | **Phenotype 4** | **Phenotype 5** |
| --- | --- | --- | --- | --- | --- | --- |
| **Burgel et al. (2010)^9^** | Number of subjects | 44 (13.7) | 89 (27.6) | 93 (28.9) | 96 (29.8) |  |
|  | Age | 58 (55-63) | 68 (60-74) | 59 (50-65) | 72.5 (67-77) |  |
|  | FEV1 % predicted | 31 (21-38) | 68 (57-76) | 46 (35-60) | 43 (33-64) |  |
|  | FVC % predicted | 63 (55-83) | 88 (78-100) | 81 (68-91) | 78 (58-92) |  |
|  | % distribution by stage GOLD1-GOLD2-GOLD3-GOLD4 | 2.2 - 0 - 47.8 - 50 | 14.6 - 70.8 - 13.5 - 1.1 | 1.1 - 41.9 - 41.9 - 15.1 | 6.2 - 34.3 - 36.5 - 22.9 |  |
|  | MMRC dyspnea score | 3 (2-4) | 1 (1-2) | 1 (1-2) | 3 (2-3) |  |
|  | Exacerbation rate | 4 (3-6) | 0 (0-1) | 1 (0-2) | 2 (1-3) |  |
|  | BMI | 19 (18-24) | 28 (25-32) | 22 (19-24) | 26 (24-30) |  |
|  | Coronary artery disease, % | 14.2 | 19.5 | 9.7 | 22.8 |  |
|  | Chronic heart failure, % | 4.7 | 12.8 | 10.8 | 35.6 |  |
|  | Diabetes, % | 0 | 17.2 | 3.2 | 19.8 |  |
|  | Hypertension, % | 19.1 | 57.5 | 20.4 | 45.7 |  |
|  | HAD scale, total | 20 (17-24) | 11 (6-14) | 12 (7-17) | 14 (11-20) |  |
|  | SGRQ, total | 70 (60-75) | 27 (19-35) | 39 (29-53) | 59 (47-67) |  |
|  | Description | Young, very severe respiratory disease, frequent exacerbator with poor nutritional status. Low prevalence of cardiovascular comorbidities. High prevalence of depression and very poor HRQoL | Old, mild respiratory disease with high prevalence of overweight. Low prevalence of cardiovascular comorbidities and depression. Mildly impaired HRQoL | Young, moderate respiratory disease with normal nutritional status. Low prevalence of cardiovascular comorbidities and depression. Moderately impaired HRQoL | Old, moderate respiratory disease, frequent exacerbator with high prevalence of overweight. High prevalence of cardiovascular comorbidities and depression. Poor HRQoL |  |
|  | Outcome analyzed- BOD score | 5 (4-6) | 1 (1-2) | 3 (2-3) | 4 (3-6) |  |
| **Burgel et al. (2012)^10^** | Outcome analyzed- crude mortality rate | 15 (35%) | 7 (8%) | 17 (20%) | 21 (25%) |  |
|  | Outcome analyzed- age at death, median, IQR | 62 (58-68) | 77 (66-83) | 67 (58-69) | 76 (74-79) |  |
|  | Age-adjusted mortality risk (Cox model) | 8.35 (3.13,22.22) *v* Phenotype 2 1.91 (0.94,3.06) *v* Phenotype 3 3.18(1.37,7.4) *v* Phenotype 4 | Reference group with lowest mortality risk | 4.33 (1.73,11.06) *v* Phenotype 2 1.67 (0.78, 3.57) *v* Phenotype 4 | 2.63 (1, 6.25) *v* Phenotype 2 |  |
| **Burgel et al. (2012)^11^** | Number of subjects | 219 (41.5) | 99 (18.8) | 209 (39.7) |  |  |
|  | Age | 62 (58-68) | 61 (57-66) | 72 (65-77) |  |  |
|  | FEV1 % predicted | 80 (65-94) | 29 (21-37) | 44 (36-58) |  |  |
|  | FVC % predicted | 105 (92-118) | 72 (61-86) | 82 (69-94) |  |  |
|  | % distribution by stage GOLD1/GOLD2/GOLD3/GOLD4 | 50/39/9/2 | 0/8/37/55 | 5/36/44/15 |  |  |
|  | MMRC dyspnea score | 0 (0-1) | 2 (1-3) | 2 (2-3) |  |  |
|  | BMI | 25 (23-28) | 20 (18-22) | 26 (24-29) |  |  |
|  | Ischemic heart disease, % | 14 | 17 | 34 |  |  |
|  | Diabetes, % | 9 | 11 | 20 |  |  |
|  | CCQ, total | 1.8 (1-3) | 6.8 (5-9) | 6.3 (4.5-8) |  |  |
|  | CT-detected emphysema, % | 48 | 96 | 81 |  |  |
|  | CT-detected Mild/moderate/severe bronchial thickening, % | 57/33/9 | 36/45/19 | 20/52/27 |  |  |
|  | CT-detected bronchiectasis, % | 14 | 31 | 33 |  |  |
|  | Description | Young, mild to moderate airflow limitation, absent or mild emphysema, absent or mild dyspnea, normal nutritional status and limited comorbidities | Young, severe airflow limitation,marked emphysema and hyperinflation, low BMI, severe dys- pnea, and impaired HRQoL. One third of the subjects were women, and osteoporosis and muscle weakness were highly prevalent, but diabetes and cardiovascular comorbidities were less prevalent. | Older, mostly male,moderate to severe airflow limitation. Less severe emphysema than subjects in Phenotype 2, but higher prevalence of bronchial thickening. Higher prevalence of obesity, diabetes and cardiovascular comorbidities. |  |  |
|  | Outcome analyzed- crude mortality rate | 1(0.5%) | 20 (20.6%) | 29 (14.3%) |  |  |
|  | Age-adjusted mortality risk (Cox model) | Reference group with lowest mortality risk | 47.5 (6.3,358.6) *v* Phenotype 1 3.3 (1.5,7.2) *v* Phenotype 3 | 14.3 (1.9,110.3) *v* Phenotype 1 |  |  |
| **Cho et al. (2010)^12^** | Number of subjects | 66 (21.4%) | 102 (33.1%) | 88 (28.6%) | 52 (16.9%) |  |
|  | Age | 66.5 | 68.6 | 66.3 | 68.2 |  |
|  | FEV_1_ % predicted | 26.5 | 34.3 | 23.9 | 26.2 |  |
|  | FVC % predicted | 67.5 | 79.1 | 62.9 | 65.2 |  |
|  | TLC, % predicted | 132.7 | 126.3 | 129.4 | 121.2 |  |
|  | Bronchodilator response, % of baseline FEV_1_ | 13.4 | 21.5 | 6.6 | 10.5 |  |
|  | UCSD Shortness of breath questionnaire score | 61.4 | 54 | 63.5 | 57.4 |  |
|  | BMI | 23.7 | 25.7 | 24.7 | 26.1 |  |
|  | 6MWD (m) | 363 | 421 | 380 | 352 |  |
|  | Maximum work (watts) | 35.5 | 53.4 | 40.9 | 40.3 |  |
|  | CT-detected total fraction emphysema at -950 HU | 0.31 | 0.13 | 0.12 | 0.11 |  |
|  | CT-detected airway wall thickness, mm | 1.36 | 1.5 | 1.45 | 1.93 |  |
|  | rs1800470 TGFB1 AA/AG/GG, % | 24/51/25 | 42/46/12 | 34/55/12 | 45/57/8 |  |
|  | Description | Emphysema predominant with a lower BMI,fewer pack-years of smoking, higher TLC, lower DLCO, lower 6MWD and maximum work | Milder severity, bronchodilator responsive, higher BMI, greater FVC and DLCO, a lower PaCO2, higher six minute walk distance and maximum work, fewer symptoms of dyspnea, and fewer exacerbations, despite being of slightly older age. | Less emphysema and lower wall thickness (similar to Phenotype 2). However, lower FEV1, less bronchodilator responsiveness, more dyspnea compared to Phenotype 2 despite a relatively younger age | Airway predominant, higher BMI, lower TLC, less severe emphysema, highest airway thickness, with a lower PaO2 and lower 6MWD |  |
|  | Outcome analyzed- exacerbations (retrospectively over 3.3 years) | 0.19 | 0 | 0.19 | 0.15 |  |
| **DiSantostefano et al. (2013)^13^** | Number of subjects | 454 (29) | 756 (49) | 333 (22) |  |  |
|  | Age, median years (IQR) | 67 (62–74) | 64 (58–70) | 65 (59–71) |  |  |
|  | Body mass index, median m/kg2 (IQR) | 28 (25–34) | 26 (23–30) | 25 (22–29) |  |  |
|  | Current smokers, % | 35 | 42 | 47 |  |  |
|  | Smoking, mean pack-years (IQR) | 52 (40-77) | 50 (37–70) | 48.5 (36–70) |  |  |
|  | FEV1% predicted (SD) | 33.9 (25.1-42.6) | 31.3 (23.9–39.4) | 37.7 (29.0–44.6) |  |  |
|  | FEV1% reversibility (SD) | 18.55 (7.4-31.7) | 26.25 (18.60–38.20) | 4.50 (-1.00–8.70) |  |  |
|  | Exacerbations requiring hospitalisation (past year) (%) – none/1/2 | 73.8/23.6/2.6 | 78.9/19.5/1.6 | 76.3/19.8/3.9 |  |  |
|  | Exacerbations requiring oral steroids/antibiotics (past year) (%) – none/1/2 | 62.6/20/8.1 | 61.1/25/7.7 | 67/19.2/7.2 |  |  |
|  | Diuretic use, % | 100 | 0 | 0 |  |  |
|  | Psycholeptic medication use, % | 32.6 | 21.4 | 24 |  |  |
|  | Anti-diabetic medication use, % | 17.4 | 7.8 | 8.4 |  |  |
|  | Anti-hypertensive medication use, % | 7.3 | 3.2 | 1.5 |  |  |
|  | Description | Treated with diuretics, having a higher BMI, with fewer current smokers, more subjects with frequent moderate exacerbations, and a higher use of cardiac medications and psycholeptics | Not treated with diuretics, having a lower FEV1  with highest FEV1 reversibility post-bronchodilator.  Fewer proportion of subjects on cardiac medications and psycholeptics | Not treated with diuretics, higher proportion of current smokers. Subjects with higher FEV1  overall, with lowest FEV1 reversibility post-bronchodilator |  |  |
|  | Outcome analyzed- response in the rate of exacerbations to SFC as compared to SAL | Reduction in the annual rate of moderate/severe exacerbations among patients randomized to SFC as compared with SAL alone (RR=0.56, p<0.001); | Reduction in the annual rate of moderate/severe exacerbations among patients randomized to SFC as compared with SAL alone (RR=0.67, p<0.001) | No change in the annual rate of moderate/severe exacerbations among patients randomized to SFC as compared with SAL alone (RR=1, p not significant) |  |  |
| **Garcia-Aymerich et al. (2011)^14^** | Number of subjects | 126 (36.9) | 125 (36.5) | 91 (26.6) |  |  |
|  | Age | 67.4 (8.8) | 68.8 (8.1) | 67.4 (9) |  |  |
|  | Height (cm) | 162 | 164 | 166 |  |  |
|  | FEV1 % predicted | 38 | 63 | 58 |  |  |
|  | TGV % predicted | 161 | 129 | 114 |  |  |
|  | MMRC dyspnea score | 3.2 | 1.9 | 2.8 |  |  |
|  | BMI | 26 | 27.2 | 32.4 |  |  |
|  | 6MWD (m) | 6 | 7 | 23 |  |  |
|  | Maximal oxygen uptake at cardiopulmonary incremental exercise test peak (% predicted) | 53 | 69 | 68 |  |  |
|  | Myocardial infacrction (doctor-diagnosed), % | 6 | 7 | 23 |  |  |
|  | Congestive heart failure (doctor-diagnosed), % | 5 | 1 | 17 |  |  |
|  | Diabetes, % | 16 | 14 | 32 |  |  |
|  | C-reactive protein (mg/dl) | 0.99 | 0.36 | 1.37 |  |  |
|  | Tumor necrosis factor (pg/ml) | 0.88 | 0.72 | 1.05 |  |  |
|  | Interleukin 6 (pg/ml) | 1.52 | 1.12 | 1.81 |  |  |
|  | CT-detected Density less than -950 Hounsfield units in both lung infracarinal thin sections (%) | 24 | 16 | 6 |  |  |
|  | CT-detected bronchial wall thickness extension in right inferior lobe (score 0 -3) | 2.7 | 1.5 | 1.1 |  |  |
|  | Description | Severe respiratory disease with poor functional capacity, emphysematous, few comorbidities | Milder respiratory disease, preserved BMI, few comorbidities | Mild respiratory disease, but high BMI, higher prevalence of comorbidities and inflammatory markers |  |  |
|  | Outcome analyzed- ATS/ERS severity stage adjusted - COPD admission risk | 2.89 (1.59 - 5.25) | Reference group with lowest mortality risk | 1.54 (0.91 - 2.63) |  |  |
|  | Outcome analyzed- ATS/ERS severity stage adjusted -Mortality | 2.01 (0.72 - 5.62) | Reference group with lowest mortality risk | 1.55 (0.67 - 3.58) |  |  |
| **Spinaci et al. (1985)^15^** | Number of subjects | 189 (36) | 343 (64) |  |  |  |
|  | Corrected FEV1, L | 0.96 (0.42) | 1.18 (0.38) |  |  |  |
|  | PaO2, mm Hg | 55.8 (7.3) | 64.9 (8.2) |  |  |  |
|  | Change in FEV1 post-bronchodilator, L | 0.09 (0.15) | 0.11 (0.14) |  |  |  |
|  | Emphysematous patients , % | 23.4 | 13.7 |  |  |  |
|  | Heavy smokers, % | 26.6 | 19 |  |  |  |
|  | Underweight patients, % | 7 | 2.1 |  |  |  |
|  | Recent hospitalizations (%) | 27.2 | 16 |  |  |  |
|  | Description | Severe respiratory disease, heavy smokers, emphysematous, frequent hospitalizations | Milder respiratory disease, preserved body weight, lower prevalence of emphysema and recent hospitalizations |  |  |  |
|  | Outcome analyzed- Analysis of contingency tables | Worse prognosis of life (details not provided) |  |  |  |  |
| **Vanfleteren et al. (2013)^16^** | Number of subjects | 67 (31.4) | 49 (23) | 44 (21) | 33 (15.5) | 20 (9.4) |
|  | Age | 62.1 (6.8) | 67.2 (5.8) | 62.5 (7.2) | 63.1 (7.3) | 62.8 (6.8) |
|  | Male, % | 60 | 65 | 43 | 79 | 45 |
|  | FEV1 % predicted | 52.7 (17.4) | 50.9 (17.7) | 48.3 (16.3) | 54.2 (16) | 48.3 (15.4) |
|  | TGV % predicted | 143 (33) | 148 (29) | 166 (34) | 134 (33) | 146 (28) |
|  | MMRC dyspnea score | 1.99 (1.01) | 2.29 (1.21) | 1.73 (0.9) | 2.12 (1.11) | 2.84 (1.12) |
|  | 6MWD (m) | 474 (102) | 446 (133) | 496 (101) | 473 (91) | 459 (74) |
|  | Number of comorbidities | 2.5 (1.4) | 3.8 (1.7) | 4.2 (1.4) | 4.4 (1.1) | 4.1 (1.8) |
|  | Obesity, % | 30 | 14 | 0 | 61 | 15 |
|  | Underweight, % | 0 | 0 | 66 | 3 | 0 |
|  | Muscle wasting, % | 12 | 10 | 98 | 0 | 20 |
|  | Osteoporosis, % | 27 | 37 | 52 | 0 | 35 |
|  | Hypertension, % | 3 | 98 | 43 | 100 | 5 |
|  | Atherosclerosis, % | 56 | 67 | 12 | 81 | 53 |
|  | Myocardial infacrction, % | 2 | 11 | 7 | 13 | 32 |
|  | Hyperglycemia, % | 52 | 41 | 43 | 91 | 60 |
|  | Dyslipidemia, % | 42 | 16 | 25 | 67 | 40 |
|  | Renal impairment, % | 16 | 24 | 45 | 9 | 5 |
|  | Anxiety, % | 5 | 28 | 26 | 0 | 84 |
|  | Depression, % | 6 | 23 | 7 | 6 | 68 |
|  | C-reactive protein (mg/dl) | 2.29 (0.84,6.19) | 3.38 (0.95,12.06) | 2 (0.68,5.94) | 3.86 (1.07,13.89) | 2.52 (0.77,8.28) |
|  | Tumor necrosis factor - R1 (pg/ml) | 2,013 (1,508, 2,689) | 2,229 (1,513, 3,285) | 1,896 (1,434, 2,505) | 2,377 (1,850, 3,055) | 2,133 (1,685, 2,699) |
|  | Tumor necrosis factor - R2 (pg/ml) | 3,417 (2,454, 4,758) | 3,698 (2,399, 5,701) | 3,302 (2,478, 4,401) | 4,080 (3,115, 5,344) | 3,419 (2,675, 4,371) |
|  | Interleukin 6 (pg/ml) | 2.4 (1.3, 4.3) | 3.4 (1.8, 6.6) | 2.2 (1.1, 4.7) | 2.7 (1.6, 4.5) | 2.2 (1.3, 3.6) |
|  | Description | Younger patients with fewer comorbidities, higher HRQoL | Older patients with a higher prevalence of cardiovascular comorbidities, poor HRQoL | Younger patients with higher prevalence of emphysema, higher prevalence of underweight, muscle wasting, osteoporosis. Over-representation of women with this phenotype. | Predominantly male, high prevalence of obesity, hyperglycemia, dyslipidemia and atherosclerosis | Younger, breathless patients with high prevalence of anxiety and depression, and poor HRQoL |
|  | Outcome analyzed- Updated BODE score | 2.4 (2.6) | 3.4 (3.3) | 3 (1.8) | 2.6 (2.3) | 3.1 (1.9) |
|  | Outcome analyzed- Framingham 10-year risk, % | 8.6 (6.6) | 11.5 (6.6) | 7.6 (6) | 11.9 (7.3) | 6.6 (4.5) |
